# Irregularities in the IARC Working Group Evaluation of Ramazzini Institute Aspartame Studies

**DOI:** 10.5334/aogh.4771

**Published:** 2025-06-06

**Authors:** Philip J Landrigan, Kurt Straif, Linda S Birnbaum, Melissa McDiarmid, Melissa J Perry, Francesco Forastiere, Pietro Comba, John R Bucher

**Affiliations:** 1 Global Observatory on Planetary Health, Boston College, Chestnut Hill, MA, USA and Centre Scientifique de Monaco, Monaco; 2 Global Observatory on Planetary Health, Connell School of Nursing, Boston College, Chestnut Hill, MA, USA, and ISGlobal, Barcelona, Spain; 3 Nicholas School of the Environment, Duke University, & National Institute of Environmental Health Sciences, National Institutes of Health, Durham, NC, USA; 4 College of Public Health, George Mason University, Fairfax, VA, USA; 5 Division of Occupational & Environmental Medicine, University of Maryland School of Medicine, Baltimore, MD, USA; 6 Department of Epidemiology, Lazio Region Health Service, Rome, IT, & Imperial College, London, UK; 7 Epidemiology Unit, Istituto Superiore di Sanità, Rome, IT; 8 National Institute of Environmental Health Sciences, National Institutes of Health, Durham, NC, USA

**Keywords:** cancer bioassays, evidence synthesis, IARC Monographs, aspartame

As members of the Ramazzini Institute International Science Advisory Board, we noted with interest the recent review of the aspartame rodent cancer data conducted by a Monographs Working Group of the International Agency for Research on Cancer (IARC) [1]. We are pleased that, in their review, the IARC Working Group considered data from a series of experimental studies on the carcinogenicity of aspartame undertaken by Soffritti and colleagues at the Ramazzini Institute [2–7].

In Section 5.3, the IARC monograph concludes that, ‘Treatment with aspartame caused an increase in the incidence of malignant neoplasms or an appropriate combination of benign and malignant neoplasms in two species (mouse and rat) and both sexes.’

The IARC monograph further states that in the Ramazzini Institute (RI) transplacental and perinatal exposure studies in mice, aspartame ‘caused hepatocellular carcinoma, hepato‑cellular adenoma or carcinoma (combined), bronchioalveolar carcinoma, bronchioalveolar adenoma or carcinoma (combined), and a significant increase in the incidence of lympho‑blastic leukaemia, monocytic leukaemia, and total myeloid tumours’ in males, and ‘in females, aspartame caused lymphoblastic leukaemia’.

The IARC monograph states additionally that, ‘in rats administered aspartame in [RI] open‑access feeding studies it “caused carcinoma and papilloma and carcinoma (combined) of the renal pelvis and ureter in males and females, and mammary gland carcinoma in females. In males, a re‑analysis of the above [study] data indicated a significant increase in the incidence of monocytic leukaemia, histiocytic sarcoma, and a positive trend in the incidence of total myeloid tumours at all doses.” In transplacental and perinatal [RI] exposure studies in male rats, aspartame “caused malignant schwannoma and in female rats, aspartame caused mammary gland carcinoma and renal pelvis papilloma. An additional re‑analysis of these data reported a positive trend for myeloid leukaemia and total myeloid tumours in females”.’

Given these strong statements by the IARC Working Group regarding the capacity of aspartame to induce cancer in experimental animals, we are baffled by the IARC monograph’s conclusion that there is only ‘limited evidence of carcinogenic activity’ of aspartame in experimental animals.

The summary of the experimental animal studies in the IARC monograph on aspartame states that, ‘Although data from the studies on transplacental and perinatal exposure followed by oral administration (feed) in mice and rats and the study by oral administration (feed) in rats indicated that aspartame had carcinogenic activity, overall the Working Group had serious questions about the adequacy of the design, conduct, interpretation and reporting of each of the studies’. For example, the Working Group stated the studies were not done under Good Laboratory Practices and that no adjustments were made for litter effects.

This summary goes on to indicate that ‘a minority of the Working Group did not consider that these concerns would substantially change the interpretation of the [sufficient] evidence of carcinogenic activity in experimental animals’. Indeed, we note that the rat lifetime study did, in fact, limit one male and one female pup per litter to each dose group [2].

We contend that the IARC aspartame Working Group failed to clearly articulate and justify its negative evaluation of the RI studies concerning the experimental carcinogenicity of aspartame. Rather than undertake a detailed examination of the design, conduct, interpretation, and reporting of the RI studies in accordance with the procedures specified in Section 3 of the IARC monograph *Cancer in Experimental Animals*, the IARC aspartame Working Group conducted a superficial, broad‑brush summary assessment that was lacking in detail and diverged sharply from the conclusions reached in the body of their analysis. We suggest that the IARC monograph would have significantly benefited from a more direct discussion of the differing opinions, including the minority opinion, and better adherence to the process for evaluations as prescribed in the Preamble to the Monograph.

Other IARC Working Groups have appropriately incorporated lifetime RI animal cancer study findings in their assessments of other chemicals [8, 9]. We submit that the IARC aspartame evaluation is an unfortunate aberration that may have been guided by factors other than dispassionate scientific evaluation of the available data.
